# Evaluation of Apically Extruded Debris During Retreatment Procedures Using Various File Systems in Teeth With Simulated Apical Root Resorption: An In Vitro Study

**DOI:** 10.7759/cureus.40904

**Published:** 2023-06-24

**Authors:** Sasti Gayatri, Sebeena Mathew, Karthick Kumaravadivel, Boopathi Thangavel, Deepa N Thangaraj, Athira Shaji

**Affiliations:** 1 Conservative Dentistry and Endodontics, KSR Institute of Dental Science and Research, Tiruchengode, IND

**Keywords:** reciproc, hyflex remover, protaper universal retreatment, root resorption, retreatment, debris extrusion

## Abstract

Aim

The aim of this study was to compare the amount of debris produced apically during the removal of root canal obturating material by using various files in extracted teeth with simulated apical root resorption.

Materials and methods

An in vitro study was conducted in the root canals of 90 extracted mandibular premolar teeth that were prepared with a ProTaper Gold rotary file (Dentsply Maillefer, Ballaigues, Switzerland) and filled with gutta-percha and an AH Plus sealer (Dentsply Maillefer, Ballaigues, Switzerland) using a cold lateral compaction technique. A total of 45 mandibular premolar teeth were randomly assigned to three control groups (i.e., the ProTaper Universal retreatment file (Dentsply Maillefer), the Reciproc Blue file (VDW, Munich, Germany), and the HyFlex Remover file (Coltene/Whaledent, Altstatten, Switzerland) for the removal of root canal filling material, whereas the remaining 45 teeth were treated as the experimental group and their apical portion was modified to simulate apical root resorption. The teeth of this experimental group were randomly divided into three subgroups according to the same three techniques used with the control groups for the removal of root canal filling materials. The apically extruded debris was collected into pre-weighed borosilicate glass tubes and then dried. The mean weight of the apically extruded debris was assessed using an analytical balance to an accuracy of 10^-4^ g. Further, the data were analyzed using the Kruskal-Wallis test and Tukey’s post hoc test.

Results

In the simulated apical root resorption groups, all file systems were associated with significantly more debris extrusion than the groups without simulated root resorption (a* *< 0.05). In both the control groups and experimental groups, the ProTaper Universal retreatment file was associated with the least weight of the apically extruded debris (a < 0.05), followed by the Reciproc Blue file and the HyFlex Remover file.

Conclusion

The amount of debris extruded apically was significantly greater in the teeth with simulated apical root resorption than in those without it. Further, during the removal of the root canal filling materials, HyFlex Remover was associated with significantly more apically extruded debris in all groups.

## Introduction

The term “root resorption” refers to the process of removing cementum and/or dentine through the physiological or pathological activity of tooth-resorbing cells called dentoclasts. The inflammatory root resorption of endodontic origin is commonly known as apical inflammatory root resorption [1]. The periradicular inflammatory response to bacterial toxins and their proteolytic enzymes induces morphological changes, which result in the resorption of the apical cementum and dentin and, consequently, expose dentinal tubules [2]. The exposed dentinal tubules act as a pathway for bacteria and their products to establish contact with the inflamed periradicular tissues, perpetuating inflammation and leading to continued dentin and cementum resorption. As a result, most teeth with apical periodontitis exhibit some degree of root resorption, which frequently goes unnoticed on radiographs and may have an impact on the clinical outcome [3].

The failure of primary endodontic treatment can be attributed to various factors such as the persistence of microorganisms as a result of insufficient biomechanical preparation, inadequate obturation, or improper coronal seal [4]. In these cases, nonsurgical endodontic retreatment is often the first choice to greatly reduce or eliminate the microbial infection in the root canal system [5]. In the available literature, various file systems and techniques have been used to remove gutta-percha from the root canal, which varies widely in the amount of debris extrusion. The extruded debris can act as a potent co-factor for interappointment flare-ups, postoperative pain, and failure or delay of periapical healing [6].

A previous study involving a root canal retreatment procedure reported that significantly more debris was extruded in a tooth with simulated apical root resorption than in a tooth without apical root resorption [7]. Thus, the incidence of postoperative pain in a tooth with apical root resorption is greater than that in a tooth with a normal apex. In the present study, the following three rotary file systems were chosen that vary in instrument kinematics and design: (i) the ProTaper Universal retreatment rotary file system (Dentsply Maillefer, Ballaigues, Switzerland), which consists of three files (D1-D3), has convex-triangular cross-sections and is manufactured specifically to perform retreatment [8]; (ii) the Reciproc Blue file system (VDW, Munich, Germany), which has an S-shaped cross-section with reciprocating motion and can be used for both primary and retreatment root canal procedures [9]; and (iii) the HyFlex Remover (Coltene/Whaledent, Altstatten, Switzerland), which is a newly introduced single rotary file system with variable triple-helix cross-section, a unique feature that is incorporated to preserve the pericervical dentin [10]. The present study was performed to compare the amount of apically extruded debris during retreatment procedures by using these three file systems in teeth with simulated apical root resorption.

## Materials and methods

Prior to the collection of the tooth samples, the sample size for the study was estimated to be 90 with a statistical power of 80%, which was calculated using the nMaster 2.0 software.

Selection and preparation of the sample

After obtaining ethical clearance from the KSR Dental Science and Research Institute (KSRIDSR) Institutional Ethics Committee as proposed by IEC-PG/FEB/2021/009, 90 mandibular single-rooted premolars with closed apices extracted for orthodontic treatment were selected for the study in accordance with the inclusion and exclusion criteria. The Preferred Reporting Items for Laboratory studies in Endodontology (PRILE) guidelines were followed.

Inclusion criteria

Non-carious intact single-rooted and single-canalled premolars with a root canal curvature of less than 10° (Schneider's method) and teeth with a minimum length of 19 mm were selected for the study.

Exclusion criteria

Premolars with immature roots, teeth with root caries, fractured roots, multiple canals, root canal calcifications, or teeth with any other developmental abnormalities were excluded from the study. The teeth were examined using an operating microscope (Lumin Pro Sanma, Chennai, India). The selected teeth were cleaned of any soft tissue remnants, calculus, and surface debris. The selected tooth samples were then autoclaved for 40 minutes and stored in the saline solution until use.

Initial instrumentation and obturation

For standardization, the tooth crowns were partially removed to achieve a final length of 19 mm. Then, standard endodontic access cavities were prepared using a round diamond bur (Mani DIA burs, Japan) at high speed along with air-water spray cooling. A size 10 K-file (Mani, Inc., MDCI Ltd., Japan) stainless steel file was used to negotiate the canal and maintain canal patency, whereas a size 15 K-file was used to establish the initial working length, which was 1 mm short of the apex. Further, the root canals were prepared using ProTaper Gold rotary files (Dentsply) up to master apical size F2 (#25). During the preparation, the canals were irrigated with 15 ml of 5.25% NaOCl (Prime Dental Products Pvt. Ltd., Thane, India). The final irrigation was completed with 5 ml of 17% ethylenediaminetetraacetic acid (EDTA), and the canals were dried with ProTaper paper points (Dentsply, Germany). A size 25, 0.06 tapered gutta-percha cone (Dentsply) was placed using an AH Plus sealer (Dentsply DeTrey, Germany). Afterward, the accessory cones were laterally compacted. Radiographs were taken to confirm adequate root filling. After root canal filling, coronal 1 mm of the root canal filling materials was removed, and the spaces were filled with temporary filling material (Cavit 3M ESPE, St. Paul, MN, USA). The filled teeth were stored at 100% humidity and 37°C for two weeks for the sealer to set [5].

Retreatment procedure and debris collection

The 45 specimens of both the control and experimental groups were randomly sub-divided into three groups (n = 15 for each group), according to the file systems used for the removal of root canal filling materials. In the control group, teeth were not modified to simulate the apical root resorption, whereas in the experimental group, the apical 2 mm of the root was removed to simulate the apical root resorption.

During the retreatment procedure, root canal fillings were removed at the original working length in the control group and the new working length in the experimental group. The retreatment procedure in both the experimental and control groups was performed using three file systems, according to the manufacturer’s instructions.

Retreatment instrumentation procedures

ProTaper Universal Retreatment Groups

At 2-Ncm torque and 500-rpm speed, root canal filling material was removed using subsequent files of D1 (size 30, 0.09 taper), D2 (size 25, 0.08 taper), and D3 (size 20, 0.07 taper). Further, the cervical third was treated with D1, the middle third with D2, and the full working length with D3. Then, the apical preparation was carried out using the ProTaper Gold F4 file (size 40, 0.06 taper) at 300 rpm in a crown-down motion [8].

Reciproc Blue File Groups

The obturated material was removed using the Reciproc Blue R25 (size 25, 0.08 taper) file using the Reciproc mode of the endodontic motor. The file was used in a gradual in-and-out pecking motion with a 3-mm amplitude limit, and gentle apical pressure was paired with a brushing motion against the lateral walls of the root canal. The final apical preparation was done using R40 (size 40, 0.06 taper) [9].

HyFlex Remover File Groups

The obturated material was removed using the HyFlex Remover (size 30, 0.07 taper) file at 2.5-Ncm torque and 500-rpm speed. The files were used with a brushing action against the canal walls in a crown-down direction. The final preparation was done using Pro Taper Gold F4 at 300 rpm [10].

Test apparatus

**Figure 1:**
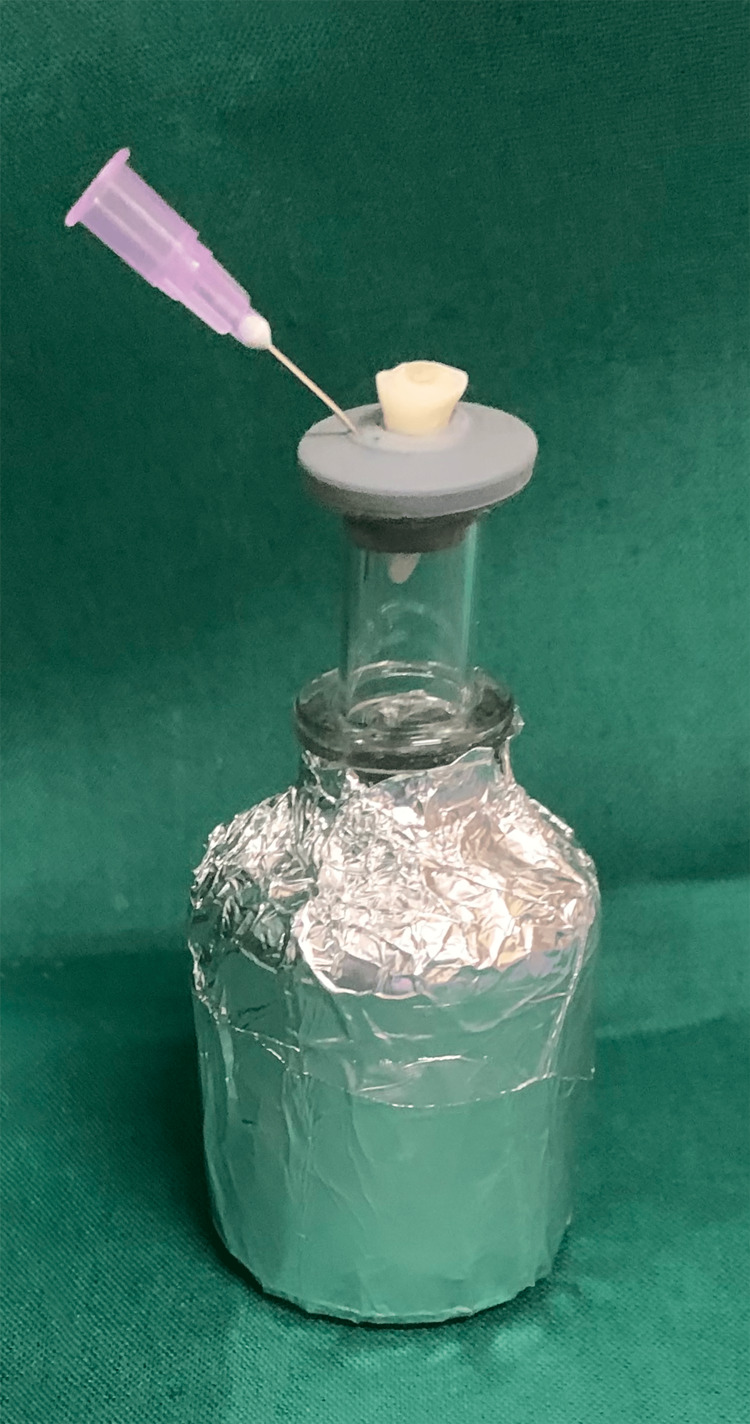
Experimental setup for collection of debris

The debris extruded during the retreatment procedure was collected using the protocol described by Myers and Montgomery [11]. It uses a borosilicate glass tube with a rubber stopper. With the help of a Bard Parker (BP) blade, a hole was cut into the rubber stopper so that the tooth could be inserted at the level of the cementoenamel junction. The root portion was held within the glass tube, which favors the collection of extruded debris during the retreatment procedure. This borosilicate glass test tube was enclosed within a glass vial to prevent contamination by the operator’s hand. Moreover, the glass vial was covered by aluminum foil to prevent the operator from viewing the debris extrusion during the instrumentation procedure. A 27G needle was inserted into the rubber stopper to normalize the air pressure inside and outside the tubes (Figure 1).

**Figure 2:**
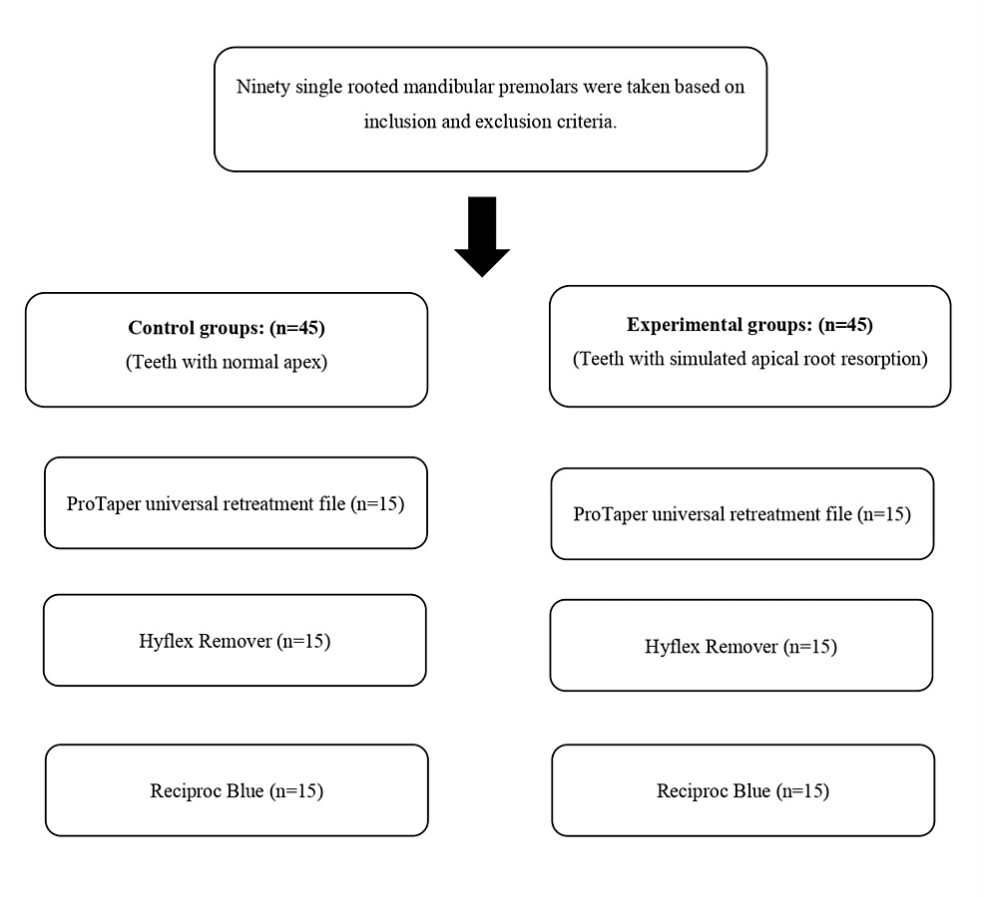
Group allocation

The borosilicate glass test tube was pre-weighed using an analytical balance (Sartorius, CP225D, Göttingen, Germany) to an accuracy of 10^-4^ g. Three consecutive measurements were taken for each borosilicate tube, and the average value was subsequently recorded. During the removal of the root canal obturating material, irrigation was performed using distilled water, which was delivered using a 30-gauge, closed-ended, single-sided, vented needle into each canal. Later, to evaporate the distilled water, the borosilicate tubes were placed in a hot air oven at 160°C for 180 minutes. The overall methodology of the study is presented in Figure 2.

The tube was weighed using the same analytical balance used previously to obtain the final weight of the tube containing the extruded debris. The dry weight of the debris was calculated by subtracting the weight of the empty tube from the weight of the tube containing the extruded debris. The data analysis was performed using the SPPS software version 20.0. The descriptive values were obtained and statistically analyzed using the Kruskal-Wallis test (non-parametric test) for both the control and experimental groups. Further, the Mann-Whitney U test (non-parametric test) was used for the intergroup comparison of the file systems, whereas the intragroup pair-wise comparison was done using Tukey’s Honestly Significant Difference (HSD) test. The level of statistical significance was kept at a < 0.05.

## Results

Table 1 illustrates the mean values and standard deviations of the amount of apically extruded debris during the removal of root canal obturating material using different file systems for both control and experimental groups.

**Table 1:** A comparison of the means of ProTaper Universal retreatment, Reciproc Blue, and HyFlex Remover between the control and the experimental groups (Mann-Whitney U test) *a < 0.05 is significant.

Study groups		Mean	Standard deviation	a-value
ProTaper Universal retreatment	Control group	0.2093	0.05861	0.001*
	Experimental group	0.4380	0.02678	
Reciproc Blue	Control group	0.2140	0.01639	0.001*
	Experimental group	0.4440	0.03376	
HyFlex Remover	Control group	0.3007	0.04935	0.001*
	Experimental group	0.5140	0.01242	

The Mann-Whitney U test revealed that all the file systems demonstrated statistically significant differences, with the amount of apically extruded debris in the experimental group being greater (a = 0.001) than in the control group (Table 1). The HyFlex remover groups were associated with the greatest weight of apically extruded debris in both control (0.3007 g) and experimental (0.5140 g) groups, followed by the Reciproc Blue group (control group: 0.2140 g; experimental group: 0.4440 g) and the ProTaper Universal retreatment group (control group: 0.2093 g; experimental group: 0.4380 g), with an a-value of 0.000 in both the control and experimental groups (Tables 2, 3).

**Table 2:** A comparison between the means of ProTaper Universal retreatment, Reciproc Blue, and HyFlex Remover in the control groups (Kruskal-Wallis test) *a < 0.05 is significant.

Control groups	N	Minimum	Maximum	Mean	Standard deviation	a-value
ProTaper Universal retreatment	15	0.04	0.27	0.2093	0.05861	0.000*
Reciproc Blue	15	0.17	0.24	0.2140	0.01639	
HyFlex Remover	15	0.19	0.37	0.3007	0.04935	

**Table 3:** A comparison between the means of ProTaper Universal retreatment, Reciproc Blue, and HyFlex Remover in the experimental groups (Kruskal-Wallis test) *a < 0.05 is significant.

Experimental groups	N	Minimum	Maximum	Mean	Standard deviation	a-value
ProTaper Universal retreatment	15	0.39	0.47	0.4380	0.02678	0.000*
Reciproc Blue	15	0.37	0.47	0.4440	0.03376	
HyFlex Remover	15	0.49	0.54	0.5140	0.01242	

In both control and experimental groups, a pair-wise intragroup comparison was done using Tukey’s post hoc test. The test revealed that the amount of apically extruded debris was significantly greater in the HyFlex Remover group than in the ProTaper Universal retreatment group or the Reciproc Blue group, with an a-value of 0.000. Moreover, no significant difference was found between the ProTaper Universal retreatment group and the Reciproc Blue group, with an a-value of 0.802 (Tables 4, 5).

**Table 4:** Pair-wise comparison between the means of ProTaper Universal retreatment, Reciproc Blue, and HyFlex Remover in the control groups (Tukey’s HSD test) *a < 0.05 is significant. HSD: Honestly Significant Difference.

Control groups		Mean difference	a-value
ProTaper Universal retreatment	Reciproc Blue	-0.00467	0.957
	HyFlex Remover	-0.09133^*^	0.000*
Reciproc Blue	ProTaper Universal retreatment	0.00467	0.957
	HyFlex Remover	-0.08667^*^	0.000*
HyFlex Remover	ProTaper Universal retreatment	0.09133^*^	0.000*
	Reciproc Blue	0.08667^*^	0.000*

**Table 5:** Pair-wise Comparison between the means of ProTaper Universal retreatment, Reciproc Blue, and HyFlex Remover in the experimental groups (Tukey’s HSD test) *a < 0.05 is significant. HSD: Honestly Significant Difference.

Experimental groups		Mean difference	p-value
ProTaper Universal retreatment	Reciproc Blue	-0.00600	0.802
	HyFlex Remover	-0.07600^*^	0.000*
Reciproc Blue	ProTaper Universal retreatment	0.00600	0.802
	HyFlex Remover	-0.07000^*^	0.000*
HyFlex Remover	ProTaper Universal retreatment	0.07600^*^	0.000*
	Reciproc Blue	0.07000^*^	0.000*

## Discussion

Endodontic retreatment procedures are performed in cases where the primary root canal therapy fails to produce favorable postoperative outcomes [12]. The primary and retreatment endodontic procedures are generally associated with the apical extrusion of pulp tissue fragments, dentine chips, necrotic tissue, microorganisms, gutta-percha, sealer, solvent, and intra-canal irrigants beyond the apical constriction [13]. This apically extruded debris is referred to as “the worm of necrotic debris,” which has a significant association with the occurrence of periapical inflammation, inter-appointment flare-ups, and postoperative pain [13,14].

It has been reported that the majority of teeth with apical periodontitis demonstrate some degree of root resorption [15]. When compared to initial root canal treatment, root canal retreatment exhibits a poor prognosis, with a low success rate that could be attributed to the extrusion of debris, especially in teeth with periapical lesions [13]. The incidence of postoperative pain in retreatment cases with apical periodontitis was found to be significantly high (13.6%) [16].

Various techniques have been used to remove gutta-percha from the root canal, including the usage of hand files, engine-driven rotary retreatment files, ultrasonic tips/files, solvents, lasers, and heat-carrying instruments [17]. From the available literature, various studies [5,18,19] have confirmed that debris extrusion is inevitable in all root canal procedures regardless of the techniques used.

Among the various methods used for the quantification of the apically extruded debris, the method provided by Myers and Montgomery [11] in 1991 has been the most widely used. In this study, distilled water was used as an irrigant instead of sodium hypochlorite solution as its tendency to crystallize could have altered the reliability of the test results and affected the weight of the apical debris extrusion [14].

The apical diameter after final preparation was standardized to size 40 in all the groups as it is evident from the literature that apical diameter has a strong association with the amount of apically extruded debris [14]. Moreover, the available literature suggests that the usage of hand files in the retreatment procedure significantly produces a greater amount of debris extrusion than the NiTi retreatment file systems [20,21]. Over the years, a variety of retreatment NiTi instruments has evolved, having overcome the limitations encountered in earlier systems. These file systems differ in their metallurgical properties, design, and kinematics, which invariably contributes to the amount of debris extrusion periapically.

The result of the current study demonstrated that there was a statistically significant difference between the amount of the apically extruded debris in teeth with or without simulated apical root resorption which was in accordance with the study by Topcuoglu et al. [7]. A possible explanation for this result could be the increased apical patency and a greater extent of debris extrusion, which is possibly related to the disruption of apical constriction by iatrogenic errors and apical root resorption [7]. Hence, based on these previous studies, it could be inferred that the apical root resorption could serve as a possible factor causing apical debris extrusion during the removal of root canal filling material and the risk of irrigant extrusion during the retreatment procedure [22,23].

In the present study, inter- and intragroup comparisons demonstrated that the ProTaper Universal retreatment file system had the least debris extrusion. In a previous study comparing the amount of debris extrusion of the ProTaper Universal retreatment file, D-RaCe, and the R-Endo retreatment file, no significant difference was found among the files that had triangular cross-sections. This finding was consistent with our own, where the ProTaper Universal retreatment file has a triangular cross-section, which could have contributed to its better performance as a triangular cross-section reduces the area of contact between the instrument and the dentin walls. During the retreatment procedure, the debris is positioned in-between the apical blade as the instrument is rotated in an auger-like fashion with an apical exertion that minimizes the amount of debris extruded apically [5].

From the result of this study, it is evident that the sequential usage of multiple files has an added advantage of maximum removal of the gutta-percha due to its greater penetration and minimal debris extrusion. This was in concurrence with a previous study by Burklein and Schäfer (2012) where multiple-file rotary instrumentation had less debris extrusion compared with reciprocating single-file systems [24].

The single-file systems, Reciproc Blue and HyFlex Remover showed greater debris extrusion, which can be due to the non-cutting tip that could have pushed the debris toward the apex [25]. Although the D2 and D3 of the ProTaper Universal retreatment file system have a non-cutting tip, they did not negatively influence the debris extrusion in our study, as it was preceded by the use of the D1 file, which has an active tip and shorter length which engages only the coronal part of the gutta-percha.

Intergroup comparison between Reciproc Blue and HyFlex Remover showed less amount of debris extrusion in the Reciproc Blue file, which was in accordance with the study by Uzun et al. [26]. In the Reciproc Blue file system, the reciprocating motion and S-shaped cross-section of the file that makes two-point contact with the canal wall have contributed to less debris extrusion than the HyFlex Remover file system which has a rotary motion and a triple-helix cross-section that makes three-point contact with the canal wall, which could have possibly attributed to the greater amount of debris extrusion in this study [27].

The main limitation of this study was that the apical resistance was not simulated. It can be stimulated using materials such as floral foam and agar gel; however, such simulation has adverse effects, such as the absorption of irrigants by foam and difficulty in stipulating a definite value of the agar gel thickness at the apex to mimic the size of the apical lesion. Another limitation of the in vitro model was that the variation in micro-hardness values of dentin may affect the results of the study. The teeth with lower hardness may extrude debris readily into the periapical tissues.

Within the limitations of the study, it can be inferred that the ProTaper Universal retreatment file system was associated with the least amount of debris extrusion periapically in conditions such as the teeth with root resorption as well as with normal apex. This retreatment system would, thus, help the clinician in reducing postoperative complications such as pain and swelling in the teeth with apical periodontitis, which was associated with a certain degree of root resorption. The root resorption can be also evident in the tooth prior to primary endodontic treatment, which was not included in this study.

## Conclusions

Under the conditions of this in vitro study, it could be concluded that there is an inadvertent extrusion of debris during the removal of root canal obturating material in all conditions, especially in teeth with simulated apical root resorption. The ProTaper Universal retreatment file system performed better with less debris extrusion compared to the Reciproc Blue and HyFlex Remover file systems in both simulated root resorption and normal conditions. Although apical debris extrusion is inevitable, clinicians should consider possible alternatives to reduce it during the retreatment procedure to reduce postoperative discomfort and enhance the success rate.
